# Effects of Novel Dinuclear Cisplatinum(II) Complexes on the Electric Properties of Human Breast Cancer Cells

**DOI:** 10.1007/s00232-013-9620-1

**Published:** 2013-12-17

**Authors:** Izabela Dobrzyńska, Elżbieta Skrzydlewska, Zbigniew A. Figaszewski

**Affiliations:** 1 Institute of Chemistry, University in Białystok, Al. Piłsudskiego 11/4, 15-443 Białystok, Poland; 2 Department of Analytical Chemistry, Medical University of Białystok, Mickiewicza 2, 15-230 Białystok, Poland; 3 Laboratory of Electrochemical Power Sources, Faculty of Chemistry, University of Warsaw, Pasteur St. 1, 02-093 Warsaw, Poland

**Keywords:** Surface charge density, Breast cancer cells lines (MDA-MB-231, MCF-7), Cisplatin, Platinum(II) complex, Lipid peroxidation

## Abstract

The aim of this study was to determine the influence of cisplatin and novel dinuclear platinum(II) complexes on the electrical properties of the membrane and the level of lipid peroxidation in the human breast cancer cell lines MDA-MB-231 and MCF-7. The basal electrical surface properties of cells are known. Changes in cell function may affect these surface properties, and those changes can be detected by electrokinetic measurements. The surface charge density of the breast cancer cell lines MDA-MB-231 and MCF-7 were measured as a function of pH. A four-component equilibrium model was used to describe the interaction between the solution ions and the breast cancer cell surface. The experimental and the theoretical charge variation curves of the breast cancer cells at pH 2.5–9 were in agreement. Measurements of the cellular malondialdehyde levels with high performance liquid chromatography were used to determine the extent of lipid peroxidation. The acid and base functional group concentrations and average association constants with hydroxyl ions were smaller in breast cancer cell membranes treated with cisplatin or novel dinuclear platinum(II) complexes compared with untreated cancer cells, and the average association constants with hydrogen ions were higher. The levels of lipid peroxidation products in breast cancer cells treated with cisplatin or novel dinuclear platinum(II) complexes were also higher than in untreated cancer cells.

## Introduction

Biological membranes are essential boundaries within living cells. They are multi-component structures that are responsible for many of the physiological functions and electrical properties of cells. Defects in any of the membrane components can be manifest as changes in electric charge as well as clinical disorders (Benga and Holmes 1984; Gennis 1989).

At the interface of the cell and the environment there is an electric bilayer. The structure and properties of the layer are determined by the components of the outer membrane layer and by the equilibrium between its components and the substances present in the environment. Any perturbations in the actions of the cell are manifested by variations in the action of the electric bilayer. An essential property of the electric bilayer is its electrical charge, which can be altered by cancerous transformation or by various drugs. For these reasons, studies on the electric charge can provide information on the equilibrium within a membrane and between the membrane and its environment, both in physiological and in nonphysiological conditions (Dobrzyńska et al. 2013; Szachowicz-Petelska et al. 2012, 2013)

Structural positive and negative charge carriers determine the electrical charge of a membrane. Positive charge carriers include free amino groups of proteins and aminophospholopids. Negative charge carriers include phospholipids, especially phosphatidylserine, sialic acid, and free carboxyl groups of proteins. Determining the electric charge of cell membranes as a function of environmental pH and determining the acid (*C*_TA_) and base (*C*_TB_) functional group concentrations, and their average association constants with hydrogen (*K*_AH_) or hydroxyl (*K*_BOH_) ions can reveal changes caused by cancer transformation and treatment with drugs (Dobrzyńska et al. 2006).

Platinum drugs are important anticancer compounds. Cisplatin is widely used for the treatment of many cancers including testicular, ovarian, bladder, cervical, head, neck, esophageal, and small cell lung cancer (Rybak and Ramkumar 2007; Giaccone et al. 2004). Cisplatin reacts with cellular components that have nucleophilic sites such as DNA, RNA, proteins, membrane phospholipids, and thiol-containing molecules. The interaction of cisplatin with genomic DNA leads to adduct formation including inter- and intra-strand DNA cross-links and DNA–protein cross-links, which inhibit replication, transcription, and translation (Capeda et al. 2007).

**Fig. 1:**
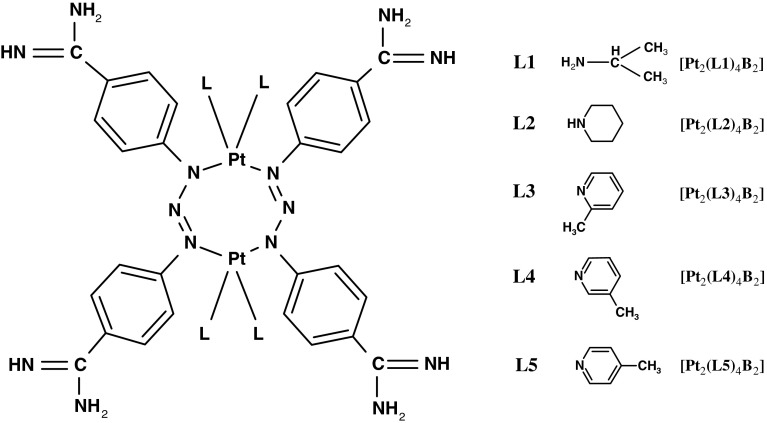
Structure of novel platinum(II) complexes

The purpose of the work is to determine the influence of cisplatin and novel dinuclear platinum(II) complexes with the structure [Pt_2_L_4_B_2_] (Fig. 1) on the electrical properties and lipid peroxidation of the cell membrane of human breast cancer cell lines MDA-MB-231 and MCF-7. Specifically, we tested platinum complexes Pt_2_(isopropylamine)_4_(berenil)_2_, [Pt_2_(L1)_4_B_2_], Pt_2_(piperazine)_4_ (berenil)_2_, [Pt_2_(L2)_4_B_2_], Pt_2_(2-picoline)_4_ (berenil)_2_, [Pt_2_(L3)_4_B_2_], Pt_2_(3-picoline)_4_ (berenil)_2_, [Pt_2_(L4)_4_B_2_], Pt_2_(4-picoline)_4_ (berenil)_2_, and [Pt_2_(L5)_4_B_2_]. The quantitative description of cell membrane properties can aid in interpreting and understanding the processes that take place on biological membranes during cancer transformation.

## Theory

The model, which has been presented in full detailed in previous study (Dobrzyńska et al. 2006), assumes that dependence of surface charge density of cell membrane on pH of electrolyte solution can be described with the help of four equilibria. There are two equilibria of negative groups, with the sodium and hydrogen ions and two equilibria of the positive groups, with the hydroxide and chloride ions. The H^+^, OH^−^ Na^+^, and Cl^−^ ions are adsorbed at the cell membrane (MDA-MB-231, MCF-7), and the adsorption equilibria (Eqs. 1–4) can by presented in the following form:

1 $${\text{A}}^{ - } + {\text{ H}}^{ + } \Leftrightarrow {\text{AH }}$$

2 $${\text{A}}^{ - } + {\text{ Na}}^{ + } \Leftrightarrow {\text{ANa}}$$

3 $${\text{B}}^{ + } + {\text{ OH}}^{ - } \Leftrightarrow {\text{BOH}}$$

4 $${\text{B}}^{ + } + {\text{ Cl}}^{ - } \Leftrightarrow {\text{BCl}}$$

The association constants of the H^+^ Na^+^, OH^−^, and Cl^−^ ions with functional groups are expressed by the following equations:

5 $$K_{\text{AH}} = \frac{{a_{\text{AH}} }}{{a_{{{{\text{A}}^{-}}}} \cdot a_{{{\text{H}}^{ + } }} }}$$

6 $$K_{\text{ANa}} = \frac{{a_{\text{ANa}} }}{{a_{{{\text{A}}^{ - } }} \cdot a_{{{\text{Na}}^{ + } }} }}$$

7 $$K_{\text{BOH}} = \frac{{a_{\text{BOH}} }}{{a_{{{\text{B}}^{ + } }} \cdot a_{{{\text{OH}}^{ - } }} }}$$

8 $$K_{\text{BCl}} = \frac{{a_{\text{BCl}} }}{{a_{{{\text{B}}^{ + } }} \cdot a_{{{\text{Cl}}^{ - } }} }}$$

where $K_{\text{AH}} ,K_{\text{ANa}} ,K_{\text{BOH}} ,K_{\text{BCl}}$—association constants, $a_{{{\text{A}}^{ - } }}$, $a_{\text{AH}}$, $a_{\text{ANa}}$, $a_{{{\text{B}}^{ + } }}$, $a_{\text{BOH}}$ and $a_{\text{BCl}}$—surface concentrations of corresponding groups on the membrane surface, $a_{{{\text{H}}^{ + } }}$, $a_{{{\text{Na}}^{ + } }}$, $a_{{{\text{OH}}^{ - } }}$ and $a_{{{\text{Cl}}^{ - } }}$—corresponding concentrations in solution.

Surface charge density (*δ*) is expressed as follows:

9 $$\delta = (a_{{{\text{B}}^{ + } }} - a_{{{\text{A}}^{ - } }} ) \cdot F$$

where $F = 96487$ $\left[ {C/{\text{mol}}} \right]$ is the Faraday constant.

Functional group concentration balances are expressed as follows:

10 $$C_{\text{TA}} = a_{{{\text{A}}^{ - } }} + a_{\text{AH}} + a_{\text{ANa}}$$

11 $$C_{\text{TB}} = a_{{{\text{B}}^{ + } }} + a_{\text{BOH}} + a_{\text{BCl}}$$

where $C_{\text{TA}}$ is the total surface concentrations of acidic groups and $C_{\text{TB}}$ is the total surface concentrations of basic groups.

Elimination of $a_{{{\text{A}}^{ - } }}$, $a_{\text{AH}}$, $a_{{{\text{B}}^{ + } }}$, and $a_{\text{BOH}}$ values from above equation yields the following formula:

12 $$\frac{\delta }{F} = \frac{{C_{\text{TB}} \cdot a_{{{\text{H}}^{ + } }} }}{{a_{{{\text{H}}^{ + } }} ( 1 { + }K_{\text{BCl}} \cdot a_{{{\text{Cl}}^{ - } }} ) + K_{\text{BOH}} \cdot K_{\text{w}} }} - \frac{{C_{TA} }}{{K_{\text{AH}} \cdot a_{{{\text{H}}^{ + } }} + K_{\text{ANa}} \cdot a_{{{\text{Na}}^{ + } }} + 1}}$$

It is difficult to solve Eq. 12 and determine the $K_{\text{AH}}$, $K_{\text{BOH}}$, $K_{\text{ANa}}$,and $K_{\text{BCl}}$ constants. In cases of high or low hydrogen ion concentrations Eq. 12 can be simplified to linear equations. In the range of high H^+^ concentration, the numerator of each term in Eq. 12 can be divided by the denominator leaving two initial terms only. These operations yield the linear equation in the $a_{{{\text{H}}^{ + } }}$ and $\frac{\delta }{F}a_{{{\text{H}}^{ + } }}$ coordinate system;

13 $$\frac{\delta }{F}a_{{{\text{H}}^{ + } }} = \frac{{C_{\text{TB}} }}{{1 + K_{\text{BCl}} \cdot a_{{{\text{Cl}}^{ - } }} }} \cdot a_{{{\text{H}}^{ + } }} - \left( {\frac{{K_{\text{BOH}} \cdot K_{\text{w}} \cdot C_{\text{TB}} }}{{(1 + K_{\text{BCl}} \cdot a_{{{\text{Cl}}^{ - } }} )^{2} }} + \frac{{C{}_{\text{TA}}}}{{K_{\text{AH}} }}} \right)$$

In graphical representation, the slope and the intercept can be easily extracted. At low H^+^ ion concentration Eq. 12 simplified to:

$$\frac{\delta }{F} = \frac{{C_{\text{TB}} \cdot a_{{{\text{H}}^{ + } }} }}{{K_{\text{BOH}} \cdot K_{\text{w}} + a_{{{\text{H}}^{ + } }} (1 + K_{\text{BCl}} \cdot a_{{{\text{Cl}}^{ - } }} )}} - \frac{{C_{\text{TA}} }}{{K_{\text{ANa}} \cdot a_{{{\text{Na}}^{ + } }} + 1 + K_{\text{AH}} \cdot a_{{{\text{H}}^{ + } }} }},$$

The numerator of each term should be divided by the denominator leaving two initial terms only. These operations yield a linear equation in the $a_{{{\text{H}}^{ + } }}^{ - 1}$ and $\frac{\delta }{F}a_{{{\text{H}}^{ + } }}^{ - 1}$ coordinate system:

14 $$\frac{\delta }{F}a_{{{\text{H}}^{ + } }}^{ - 1} = \frac{{ - C_{\text{TA}} \cdot a_{{{\text{H}}^{ + } }}^{ - 1} }}{{1 + K_{\text{ANa}} \cdot a_{{{\text{Na}}^{ + } }} }} + \left( {\frac{{C_{\text{TB}} }}{{K_{\text{BOH}} \cdot K_{\text{w}} }} + \frac{{K_{\text{AH}} \cdot C{}_{\text{TA}}}}{{(1 + K_{\text{ANa}} \cdot a_{{{\text{Na}}^{ + } }} )^{2} }}} \right)$$

In graphical representation, the slope and the intercept can be easily extracted.

The coefficients estimated from the linear regression can be used to determine $C_{\text{TA}}$, $C_{\text{TB}}$, $K_{\text{AH}}$, and $K_{\text{BOH}}$. The points included in the regression must be carefully selected, both in high and low pH ranges. Defining the value of these parameters permits the calculation of the theoretical cell membrane surface charge from Eq. 12 for comparison to experimental date.

## Materials and Methods

### Cell Culture

The human breast cancer cell lines MCF7 and MDA-MB231 (derived by the American Type Culture Collection) were maintained in Dulbecco’s modified Eagle’s medium containing 10 % fetal bovine serum, 50 U/ml penicillin, and 50 μg/ml streptomycin. Cells were cultured in a humidified atmosphere with 5 % CO_2_ at 37 °C. The cells reached confluence after three days and were used for the assays.

Cell suspensions, 1 × 10^6^ cells/ml in 6 ml of culture medium, were incubated with or without the experimental compounds in cell culture plates. The platinum(II) complexes [Pt_2_(L1)_4_B_2_, Pt_2_(L2)_4_B_2_, Pt_2_(L3)_4_B_2_, Pt_2_(L4)_4_B_2_, and Pt_2_(L5)_4_B_2_] and cisplatin were added to the cultured cells to give a final concentration 20 μM. Control cells were incubated without test compounds. Cells from each cell line were harvested after 12 h of incubation.

### Lipid Peroxidation

The extent of lipid peroxidation in cells was assayed by measuring malondialdehyde (MDA) levels. MDA levels were estimated by performing a condensation reaction with thiobarbituric acid (TBA) with MDA. The products were separated by high performance liquid chromatography (HPLC). In brief, 0.75 ml phosphate acid solution (0.44 M) and 0.25 ml freshly prepared TBA solution (42 mM) were added to 0.5 ml diluted cells, and the mixture was incubated for 60 min at 100 °C. After the mixture cooled, 0.5 ml was neutralized with 0.5 ml 1 M methanol–1 M NaOH (45.5:4.5; v:v). After centrifugation, 30 μl of the solution was injected into the chromatographic column (RP18). Separation was carried out with an isocratic elution of 40 % methanol and 60 % phosphate buffer pH 7.0. Detection was performed with a spectrofluorometric detector (*λ*_excitation_ = 532 nm; *λ*_emission_ = 553 nm). The concentration of MDA was expressed in nmoles TBA-rs/ml in the cell lysates.

### Electrochemical Method

In order to determine the surface charge density of cell membranes, cells were suspended in 0.015 M NaCl in a measuring vessel, and electrophoretic mobility was measured using Zetasizer Nano ZS apparatus (Malvern Instruments). The measurements were carried out as a function of pH. The surface charge density was determined by the equation: *σ* = *ηu*/*d* in which *u* was the electrophoretic mobility, *η* was the viscosity of the solution, and *d* was the diffuse layer thickness (Krysiński and Tien 1986).

The diffuse layer thickness was determined from the formula (Barrow 1996) $d = \sqrt {\frac{{\varepsilon \cdot \varepsilon_{0} \cdot R \cdot T}}{{2 \cdot F^{2} \cdot I}}}$, where *R* was the gas constant, *T* was the temperature, *F* was the Faraday number, *I* was the ionic strength of 0.9 % NaCl, and *εε*_o_ was the permeability electric medium.

### Statistical Analysis

The data obtained in this study were expressed as mean ± SD. The data were analyzed using one-way ANOVA with Scheffe’s *F* test for multiple comparisons. The values for *P* < 0.05 were considered significant.

## Results and Discussion

Chemotherapy plays an important role in the treatment of breast cancer, a leading cause of cancer death among women. This study was conducted to examine the effects of new chemotherapeutic complexes on the electrical properties and extent of lipid peroxidation in human breast cancer cells.

**Fig. 2:**
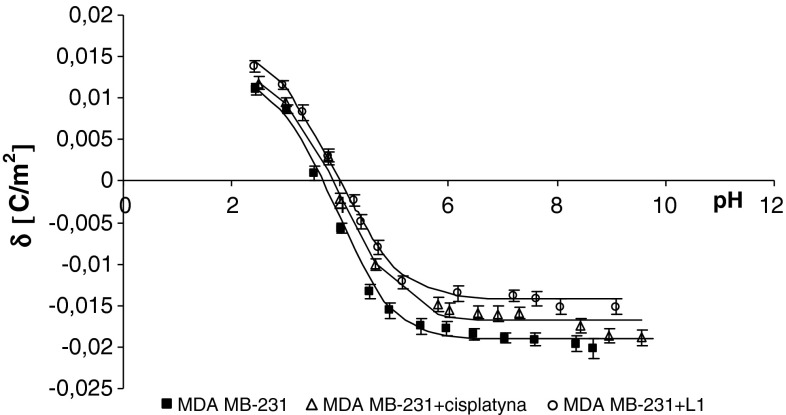
The membrane charge density of MDA-MB-231 breast cancer cells with and without treatment with cisplatin and Pt_2_(L1)_4_B_2_. The experimental values are marked by *points* and the theoretical ones by *line*

**Fig. 3:**
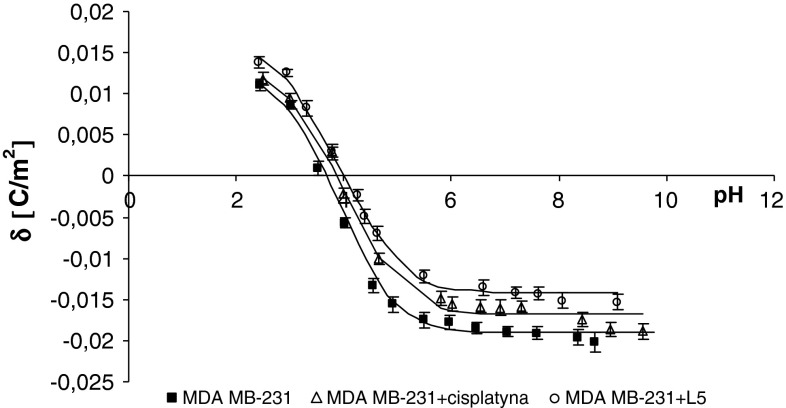
The membrane charge density of MDA-MB-231 breast cancer cells with and without treatment with cisplatin and Pt_2_(L5)_4_B_2_. The experimental values are marked by *points* and the theoretical ones by *line*

**Fig. 4:**
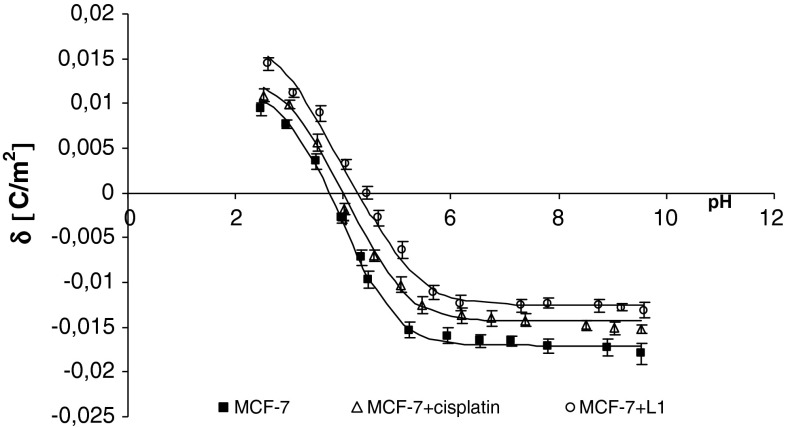
The membrane charge density of MCF-7 breast cancer cells with and without treatment with cisplatin and Pt_2_(L1)_4_B_2_. The experimental values are marked by *points* and the theoretical ones by *line*

The experimental and theoretical surface charge densities of the cell membrane measured as a function of pH are presented in Figs. 1, 2, 3, and 4. The experimental measurements were indicated by points, and the theoretical values were represented as curves. The surface charge density dependencies of MDA-MB and MCF-7 cells on pH produced similarly, shaped curves for all studies.

**Fig. 5:**
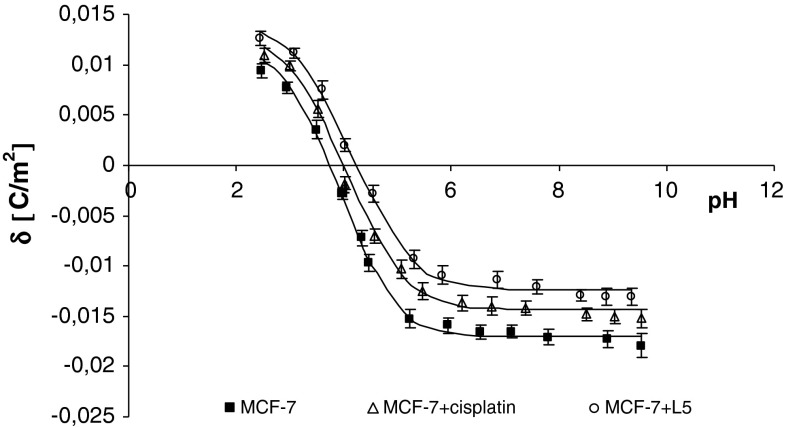
The membrane charge density of MCF-7 breast cancer cells with and without treatment with cisplatin and Pt_2_(L5)_4_B_2_. The experimental values are marked by *points* and the theoretical ones by *line*

The treatment of MDA-MB cells with cisplatin or novel dinuclear platinum(II) complexes caused a decrease in negative charge at high/low pH values compared with untreated cells (Figs. 2, 3). The isoelectric point of MDA-MB cell membranes treated with cisplatin or novel dinuclear platinum(II) complexes shifted to higher pH values compared with untreated MDA-MB cell membranes. Similarly, administering cisplatin or novel dinuclear platinum(II) complexes to MCF-7 cells caused a decrease in negative charge at high pH values and an increase in positive charge at low pH values compared with untreated MCF-7 cells (Figs. 4, 5). The isoelectric point of cisplatin or novel dinuclear platinum(II) complex treated MCF-7 cell membranes shifted to higher pH values compared with untreated MCF-7 cell membranes.

Mathematical calculations based on the four equilibria model described the adsorption of electrolyte ions on a cell surface membrane. We used this to enable quantitative evaluations of the membrane parameters. The total concentrations of functional *C*_TA_ and *C*_TB_ groups was determined for treated and untreated breast cancer cell lines MDA-MB and MCF-7. The average association constants *K*_AH_ and *K*_BOH_ were calculated based on Eqs. 13 and 14. The *C*_TA_, *C*_TB_, *K*_AH_, and *K*_BOH_ constants resulting from the calculations were substituted into Eq. 12 yielding the theoretical curve. Experimental points and theoretical curves are presented in Figs. 1, 2, 3, and 4. The theoretical and experimental surface charge density values agreed.

The experimental results indicated that cisplatin or novel dinuclear platinum(II) complexes caused a decrease in negative charge at the MDA-MB cell surface. This corresponded to a decreased surface concentration of *C*_TA_ groups. Changes in functional group composition of the MDA-MB cell membrane can be due to the appearance or disappearance of new functional groups resulting from the reactions with cisplatin or novel dinuclear platinum(II) complexes. Treatment with cisplatin or novel dinuclear platinum(II) complexes increased the association constants of negatively charged *K*_AH_ and decreased the association constants of positively charged *K*_BOH_ groups (Table 1).

**Table 1:** Effects of cisplatin and novel dinuclear platinum(II) complexes on *C*_TA_, *C*_TB_, *K*_AH_, and *K*_BOH_ of MDA-MB-221 breast cancer cells

Groups	Parameters			
	*C*_TA_ (10^−7^ mol/m^2^)	*C*_TB_ (10^−7^ mol/m^2^)	*K*_AH_ (m^3^/mol)	*K*_BOH_ (10^7^ m^3^/mol)
MDA-MB-231	1.90 ± 0.08	1.31 ± 0.06	18.80 ± 1.08	4.97 ± 0.18
MDA-MB-231 + cisplatin	1.58 ± 0.04^a^	1.48 ± 0.05^a^	22.81 ± 1.10^a^	3.97 ± 0.12^a^
MDA-MB-231 + [Pt_2_(L1)_4_B_2_]	1.43 ± 0.07^a,b^	1.63 ± 0.06^a,b^	32.80 ± 1.10^a,b^	3.62 ± 0.19^a,b^
MDA-MB-231 + [Pt_2_(L2)_4_B_2_]	1.37 ± 0.10^a,b^	1.69 ± 0.08^a,b^	35.41 ± 1.11^a,b^	3.54 ± 0.12^a,b^
MDA-MB-231 + [Pt_2_(L3)_4_B_2_]	1.48 ± 0.10^a^	1.51 ± 0.09^a^	28.89 ± 1.10^a,b^	3.81 ± 0.11^a^
MDA-MB-231 + [Pt_2_(L4)_4_B_2_]	1.41 ± 0.10^a,b^	1.59 ± 0.10^a^	30.72 ± 1.10^a,b^	3.65 ± 0.11^a,b^
MDA-MB-231 + [Pt_2_(L5)_4_B_2_]	1.45 ± 0.06^a,b^	1.62 ± 0.05^a,b^	32.46 ± 1.10^a,b^	3.67 ± 0.10^a,b^

*p* < 0.05

^a^Compared with cancer cell (MDA-MB-231)

^b^Compared with cisplatin

The experimental results indicate that cisplatin (new complexes) caused a decrease in negative charge numbers at the MCF-7 cell surface. This corresponded to a decrease surface concentration of *C*_TA_ and an increased surface concentration of *C*_TB_. Similar to MDA-MB cells, changes in functional group composition on the membrane surface was due to the appearance or disappearance of functional groups on the MCF-7 cell surface. Cisplatin or new complexes increased the association constants of negatively charged *K*_AH_ and decreased the association constants of positively charged *K*_BOH_ groups (Table 2). Higher changes were observed after treatment with novel dinuclear platinum(II) complexes compared with cisplatin.

**Table 2:** Effects of cisplatin and novel dinuclear platinum(II) complexes on *C*_TA_, *C*_TB_, *K*_AH_, and *K*_BOH_ of MCF-7 breast cancer cells

Groups	Parameters			
	*C*_TA_ (10^−7^ mol/m^2^)	*C*_TB_ (10^−7^ mol/m^2^)	*K*_AH_ (m^3^/mol)	*K*_BOH_ (10^7^ m^3^/mol)
MCF-7	1.73 ± 0,01	1.16 ± 0,09	26.81 ± 1.10^a^	2.92 ± 0.11^a^
MCF-7 + cisplatin	1.43 ± 0.50^a^	1.31 ± 0.05^a^	32.80 ± 1.11^a^	3.17 ± 0.10^a^
MCF-7 + [Pt_2_(L1)_4_B_2_]	1.25 ± 0.06^a,b^	1.71 ± 0.06^a,b^	48.47 ± 1.10^a,b^	3.82 ± 0.12^a,b^
MCF-7 + [Pt_2_(L2)_4_B_2_]	1.31 ± 0.05^a,b^	1.54 ± 0.05^a,b^	38.48 ± 1.12^a,b^	3.98 ± 0.14^a,b^
MCF-7 + [Pt_2_(L3)_4_B_2_]	1.30 ± 0.06^a,b^	1.59 ± 0.06^a,b^	37.52 ± 1.15^a,b^	3.93 ± 0.16^a,b^
MCF-7 + [Pt_2_(L4)_4_B_2_]	1.32 ± 0.05^a,b^	1.60 ± 0.07^a,b^	40.41 ± 1.11^a,b^	3.87 ± 0.12^a,b^
MCF-7 + [Pt_2_(L5)_4_B_2_]	1.32 ± 0.04^a,b^	1.61 ± 0.04^a,b^	39.54 ± 1.16^a,b^	3.92 ± 0.11^a,b^

*p* < 0.05

^a^Compared with cancer cell (MCF-7)

^b^Compared with cisplatin

Table 3 shows levels of the lipid peroxidation product MDA measured by HPLC. The treatment of MDA-MB-231 cells with cisplatin or the cisplatin complexes caused an increase in the MDA level compared with untreated cancer cells. Similarly, the treatment of MCF-7 cells with cisplatin or other cisplatin complexes caused an increase in the MDA level compared with untreated cancer cells.

**Table 3:** Effect of cisplatin and novel dinuclear platinum(II) complexes on the lipid peroxidation products measured as MDA in the breast cancer cell lines MDA-MB-231 and MCF-7

Groups	MDA-MB-231	MCF-7
Control	1.25 ± 0.04	10.32 ± 0.43
Cisplatin	1.43 ± 0.04^a^	13.75 ± 0.51^a^
[Pt_2_(L1)_4_B_2_]	1.86 ± 0.05^a,b^	9.82 ± 0.41^b^
[Pt_2_(L2)_4_B_2_]	2.47 ± 0.08^a,b^	8.95 ± 0.36^a,b^
[Pt_2_(L3)_4_B_2_]	1.43 ± 0.05^a^	15.43 ± 0.67^a,b^
[Pt_2_(L4)_4_B_2_]	2.04 ± 0.05^a,b^	8.29 ± 0.37^a,b^
[Pt_2_(L5)_4_B_2_]	2.30 ± 0.08^a,b^	10.12 ± 0.45^b^

*p* < 0.05

^a^Compared with cancer cell (MDA-MB-231 or MCF-7)

^b^Compared with cisplatin

Reactive oxygen species (ROS) have been reported to play an important role in apoptosis by regulating the activity of certain enzymes involved in the cell death pathway. Many anticancer agents have been found to induce cancer cell apoptosis by increasing ROS (Chen et al. 2008). Growing evidences suggest that generation of ROS is an important cellular event induced by chemotherapeutic drugs. Nonpublished our data shown significant increase in ROS level in the breast cancer cells after berenil–platinum(II) complexes. Moreover, it was shown that berenil–platinum(II) complexes are more potent antiproliferative agents than cisplatin. The degree to which these compounds inhibited cell growth breast cancer cells was generally consistent with their relative DNA-binding affinity, and detection of apoptosis by a fluorescent microscopy assay revealed that novel dinuclear platinum(II) complexes inhibited the proliferation of MCF-7 breast cancer cells by increasing the number of apoptotic and necrotic cells (Bielawski et al. 2010; Poplawska et al. 2009). These complexes influence cellular metabolic pathways through the expression of transcription factor NFkB and in consequence increased expression MAPkinases (ERK1/2, p38) and IGF-1 and B1 integrin receptor (Bielawska et al. 2010).

Cell membrane charge is affected by sialic acid present on glycolipids and glycoproteins and approximately 70 % of total sialic acid is found on the cell surface. It has been proposed that sialic acid also influences the surface concentration of *C*_TA_ and *C*_TB_ groups, as well as association constants with the positive and negative groups during cancer transformation and after drug treatment. The literature indicates that during cancer transformation, sialic acid content on glycolipids and glycoproteins increases (Oliva et al. 2006; Weigelt et al. 2005; Narayanan 1994). The decrease of sialic acid on cancer cells after of cisplatin or other drug treatments may be associated with an enhanced immune response of the host. It has been suggested that the loss of sialic acid decreases the surface concentration of *C*_TA_ groups and may lead to increased cell deformity and enhanced susceptibility to phagocytosis (Nicol and Prasad 2002).

Platinum(II) complexes preferentially attack membrane proteins. These complexes can also interact with phospholipids, but the bonds are relatively weak and reversible (Gea Speelmans et al. 1996; Chu 1994). The unstable interaction with phospholipids provokes changes in phospholipid conformation and in the structure and permeability of the membranes. The bond between platinum and monomeric proteins also provokes conformation changes and perturbs self-association of the monomers. The binding of platinum(II) complexes to the actin may be one of the reasons causes cell death by cross-linking and aggregating monomers, which depolymerizes microfilaments (Szachowicz-Petelska et al. 2001). Literature date show that at the molecular level, actin is indeed remodeled considerably in cisplatin-sensitive and cisplatin-resistant cells (Sharma et al. 2012). The increase in the number of apoptotic and necrotic cells after dinuclear platinum(II) complexes confirm this suggestion, but the reason may be also connected with changes in metabolic pathway through the affect NFkB signaling (Poplawska et al. 2009).

Proteins bound to DNA react with membrane phospholipids. Their activity in DNA replication, transcription, and recombination is modified by acidic phospholipids (Sekimizu 1994). It is suggested that the changes in membrane composition are connected with the changes in cell membrane charge. In conclusion, new platinum(II) complexes disturb the electrical properties and lipid peroxidation of cell membranes more effectively than cisplatin.

The constants *C*_TA_, *C*_TB_, *K*_AH_, and *K*_BOH_ are suitable for monitoring the changes caused by platinum(II) drugs. Therefore, evaluating the effects of anticancer drugs on the cell membrane may be an important component in future studies of cancer cell biology.
